# Advancing the development of TRIP13 inhibitors: A high-throughput screening approach

**DOI:** 10.1016/j.slasd.2025.100233

**Published:** 2025-04-12

**Authors:** Rae M. Sammons, Soma Ghosh, Lacin Yapindi, Eun Jeong Cho, Faye M. Johnson, Kevin N. Dalby

**Affiliations:** a Targeted Therapeutic Drug Discovery & Development Program, The University of Texas at Austin, Austin, TX, United States; b Thoracic, Head and Neck Medical Oncology, The University of Texas, MD Anderson Cancer Center, Houston, TX, United States; c Division of Chemical Biology & Medicinal Chemistry, College of Pharmacy, The University of Texas at Austin, Austin, TX, United States

**Keywords:** TRIP13, ATPase, Rb-deficient cancers, Luminescence, High throughput compound screening

## Abstract

TRIP13, a promising target for cancer therapy, has been identified as a key regulator of the mitotic checkpoint. Overexpression of TRIP13 is associated with poor clinical outcomes in various cancers. Inhibition of TRIP13 has the potential to address therapeutic challenges in cancer, particularly in therapy-resistant and Rb-deficient cancers. Despite the potential therapeutic benefits of TRIP13 inhibition, the development of TRIP13 inhibitors has been hindered by the lack of a robust high-throughput screening (HTS) assay.

We developed a luminescence-based biochemical assay for TRIP13 activity to address this challenge using the ADP-Glo detection system. This assay offers high sensitivity, low background signal, and ease of automation, making it ideal for HTS applications. A pilot screen of kinase-focused inhibitors library and a large-scale screen of 4000 additional compounds demonstrated the assay’s robust performance with a z’-factor exceeding 0.85 and a signal-to-background (S/B) ratio near 6. From the 50 initial hits, rigorous validation identified anlotinib as the most potent TRIP13 inhibitor with an IC_50_ of 5 μM. A cellular thermal shift assay (CETSA) confirmed the direct binding of anlotinib to TRIP13, validating the potential of our biochemical assay for identifying novel TRIP13 inhibitors. Our study provides a valuable tool for discovering novel TRIP13 inhibitors and advances our understanding of the therapeutic potential of targeting TRIP13 in cancer.

## 1. Introduction

Thyroid hormone receptor interactor 13 (TRIP13) plays a critical role in mitosis and has emerged as a potential target for cancer therapy. TRIP13 is an AAA+ ATPase that catalyzes ATP-dependent Mad2 unfolding, converting closed Mad2 (C-Mad2, active) to open Mad2 (O-Mad2, inactive) with p31^comet^ (*MAD2L1BP*) serving as an adaptor protein that recruits TRIP13 to C-Mad2 [1]. Mad2 is part of the mitotic checkpoint complex (MCC) that regulates the spindle assembly checkpoint (SAC). The SAC inhibits anaphase progression until all sister chromatids are properly attached to spindle poles by microtubules bound to kinetochores. In cancer cells, one potential mechanism to prevent Mad2-mediated G1 arrest and mitotic cell death [2,3], is regulating Mad2 function via TRIP13. Cancer cells overexpressing Mad2 have an increased dependency on TRIP13 for their mitotic exit [4].

TRIP13 inhibition may be useful in treating diverse cancer types, including hepatocellular carcinoma (HCC) [5–7], myeloma [8], colon cancer [9–12], chronic lymphocytic leukemia (CLL) [13], bladder cancer [14,15], head and neck squamous cell carcinoma (HNSCC) [16], non-small cell lung cancer (NSCLC) [17], and prostate cancer [18]. TRIP13 is overexpressed in these cancers, which correlates with poor clinical outcomes [19]. TRIP13 inhibition may also be effective in combination with other therapeutics. We demonstrated that the combination of Aurora kinase A inhibition and TRIP13 depletion causes extensive apoptosis selectively in retinoblastoma (RB1, Rb) deficient cancer cells, sparing Rb-proficient and non-transformed cells [20]. This cancer cell-specific effect allows for reduced doses of Aurora A inhibitors, potentially leading to a wide window of therapeutic efficacy with low human toxicity. Notably, several Rb-deficient squamous cancer cell lines are resistant to standard therapies, including cisplatin and radiotherapy, suggesting the potential effectiveness of this combination strategy in treating recurrent and therapy-resistant cancers, a critical area of unmet need. While Aurora A inhibitors are already under clinical investigation, there are currently no TRIP13 inhibitors in clinical development. This emphasizes the need to develop potent and selective TRIP13 inhibitors to complement existing therapies and address therapy-resistant cancers.

Inhibitor discovery can be achieved through various methods, each with advantages and limitations. Virtual screening, particularly when the target’s structure is known, offers a valuable approach to identifying potential inhibitors without extensive experimental screening. DCZ0415 [21], the first reported small-molecule inhibitor targeting TRIP13′s active site [22], illustrates inhibitor discovery through virtual screening. Subsequent studies focused on developing analogs, including DCZ5417 [23], DCZ5418 [24], and TI17 [25] to improve inhibitory potency. This effort highlights the growing interest in finding potent TRIP13 inhibitors. Despite these advancements, no TRIP13 inhibitors have yet progressed to clinical development. Additionally, these compounds often exhibit weak activity. For example, the biological effects of DCZ0415 in cancer cells often require concentrations of greater than 10 μM. While the binding of DCZ0415 to TRIP13 was demonstrated by a pull-down assay, as well as by nuclear magnetic resonance spectroscopy and surface plasmon resonance, these assays did not reveal the mechanism of binding [7,12,21,26]. Additionally, neither the inhibition of the TRIP13 ATPase activity nor target inhibition was demonstrated in cells. Similar findings were reported for DCZ5417, DCZ5418, and TI17.

Notably, DCZ0415 and TI17 were identified via virtual screen, while DCZ5417 and DCZ5418 were derived from natural products. While virtual screening has yielded promising leads, biochemical high-throughput screening (HTS) remains critical for identifying potent and selective inhibitors. HTS allows for the rapid evaluation of large compound libraries, enabling the discovery of novel chemical scaffolds and optimizing lead compounds. A robust and cost-effective HTS assay is essential for successfully implementing HTS campaigns. This study addresses a critical gap in developing TRIP13 inhibitors by developing and optimizing an HTS-compatible assay for TRIP13 activity. The assay utilizes the ADP-Glo detection system, which offers several advantages for inhibitor screening, including high sensitivity, low background signal, and ease of miniaturization and automation [27,28]. Following assay development, we demonstrate its high-throughput small molecule screening capability. We then validate the identified hits from the screening process using a series of biochemical and cellular assays. This optimized assay paves the way for identifying novel and potentially more potent TRIP13 inhibitors, ultimately contributing to developing new therapeutic strategies for cancer.

## 2. Materials and methods

Full length human TRIP13 (H00009319-P01, 73.26 kDa, 1–432 aa, N-GST-tagged, i.e., TRIP13) and truncated human TRIP13 (513,912, 59 kDa, 1–301 aa, N-GST-tagged, i.e., TRIP13-TR) were purchased from Abnova and NovoPro Bioscience Inc, respectively. Assay buffer contains 25 mM Tris–HCl, pH 7.5, 100 mM NaCl, 20 mM MgCl_2_, 1 mM DTT, 5 % (w/v) glycerol, and 0.02 % (w/v) Tween 20. ADP-Glo Kinase Assay kit was purchased from Promega (V6930). The HeLa cells were obtained, maintained, and profiled as previously described [20]. Anti-TRIP13 antibody (TRIP13 A-7, sc-514,285) was obtained from Santa Cruz Biotechnology, Inc. Anlotinib and DCZ0415 were purchased from Selleckchem or TargetMol and used as received.

### 2.1. Luminescence-based assay optimization

#### General TRIP13 ATPase activity assay:

Reactions were initiated by adding 5 μL of 2X ATP to 5 μL of 2X TRIP13. Reactions were conducted in 384-well assay plates (Corning 3825) with a volume of 10 μL per well. Controls included 10 μM ATP (0 % conversion), 1 μM ADP with 9 μM ATP (10 % conversion), and 10 μM ADP (100 % conversion) when 10 μM ATP was used in the assay. For assays using 5 μM ATP, the control concentrations were adjusted accordingly. ATPase reactions and controls were quenched with ADP-Glo Reagent (Glo 1, 10 μL per well) from the ADP-Glo Kinase Assay kit and incubated at room temperature for 40 min. Then Kinase Detection Reagent (Glo 2, 20 μL) from the ADP-Glo Kinase Assay kit was added to each well and incubated at room temperature for 30 min. After this step, a luminescence signal was detected using a Synergy Neo2 plate reader (Agilent). After each mixing step described above, plates were centrifuged at 800 rpm for 2 min. Both Glo 1 and Glo 2 were supplemented with 0.01 % (w/v) Tween 20.

#### Assay component and condition optimization:

1) TRIP13 concentration: TRIP13 (or TRIP13-TR) at varied concentrations (0, 50, 100, and 200 nM) was reacted with 10 μM ATP in an assay buffer for 90 min at 37 °C. 2) ATP concentration: TRIP13 (100 nM) was reacted with varied concentrations of ATP (0–100 μM) for 3 h at room temperature. 3) Room temperature stability: TRIP13 (100 nM) was reacted with 5 μM ATP for 90 min for 3 h at room temperature. 4) Assay tolerance to buffer additives: TRIP13 (100 nM) was reacted with 5 μM ATP in the presence of 0–5 % (v/v) DMSO or 0–0.5 % (w/v) Tween 20. In detail, 1 μL of 10X DMSO or Tween 20 was added to 4 μL of 2.5X TRIP13, and reactions were initiated with 5 μL of 2X ATP. 5) ADP-Glo Kinase Assay reagents: Samples containing 5 μM ADP in 10 μL volumes were tested with varying volumes of Glo 1 and Glo 2 at a 1:2 volumetric ratio. The volumes ranged from 5 μL/10 μL to 10 μL/20 μL of Glo 1/Glo 2 reagents, following the activity assay procedure.

### 2.2. Luminescence-based assay validation and compound screening

First, 10 nL of compounds (or DMSO for controls) were dispensed into assay plates using an Access and Echo 550 liquid handler (Beckman Coulter). Second, 5 μL of 2X TRIP13 (100 nM final for test samples and negative controls) or assay buffer (0 nM TRIP13 for positive controls) was dispensed using a Microflo FX liquid dispenser (Agilent). Plates were centrifuged at 800 rpm for 2 min. After 30 min of incubation at room temperature, 5 μL of 2X ATP (5 μM final) was dispensed to all wells in each plate using the Microflo FX. Plates were again centrifuged at 800 rpm for 2 min, sealed, and incubated at room temperature for 3 h. Third, 5 μL of Glo 1 was dispensed to all wells in each plate using the Microflo FX. Plates were again centrifuged at 800 rpm for 2 min and incubated at room temperature for 40 min. Fourth, 10 μL of Glo 2 was dispensed to all wells in each plate using the Microflo FX. Plates were centrifuged at 800 rpm for 2 min and incubated at room temperature for 30 min. Luminescence signals were then detected using a Synergy Neo2 plate reader (Agilent).

### 2.3. Hit compound validation

#### Rescreen and counter-screen:

Selected compounds were retested at 10 μM using the same assay conditions as the screening procedure. Compounds or DMSO controls were dispensed using the Echo liquid handler, and all other reagents were dispensed by manual pipetting. Compounds were subjected to a counter-screen assay to assess potential assay interference. In this counter-screen, TRIP13 and ATP were replaced with 0.5 μM ADP in the assay buffer, maintaining the same assay conditions as the primary screen.

#### Compound dose-response assays:

TRIP13 (150 nM) was reacted with 5 μM ATP in the presence of varying concentrations of compounds (0–200 μM). In detail, 1 μL of 10X compound was added to 4 μL of 2.5X TRIP13, and reactions were initiated with 5 μL of 2X ATP. The counter-screen included 0–200 μM compounds with 0.5 μM ADP. All reactions were normalized to contain 2 % (v/v) DMSO, corresponding to the maximum concentration of 200 μM. For DCZ0415, TRIP13 at a final concentration of 100 nM was used, DCZ0415 dilution started from 500 μM, and all reactions were normalized to a control containing 5 % (v/v) DMSO.

#### Cellular Thermal Shift Assay (CETSA):

CETSA was conducted following a modified protocol based on established methods [29]. HeLa cells were incubated with anlotinib or DMSO for 6 h. Subsequently, the treated cells were collected, rinsed with chilled PBS, and suspended in a lysis buffer containing 25 mM Tris–HCl (pH 7.5), 10 mM MgCl_2_, and a complete protease inhibitor cocktail. The cell suspension was divided into 100 μL aliquots in PCR tubes and subjected to a temperature gradient (25, 55, 64, 67, 70, and 74 °C) for 4 min each. The samples underwent three freeze-thaw cycles using liquid nitrogen to ensure complete lysis. The lysates were then centrifuged at 20,000 × *g* for 20 min at 4 °C. Protein content in the resulting supernatants was quantitated and analyzed using western blotting techniques.

## 3. Results and discussion

### 3.1. Optimization of a luminescence-based TRIP13 ATPase assay

**Fig. 1.**
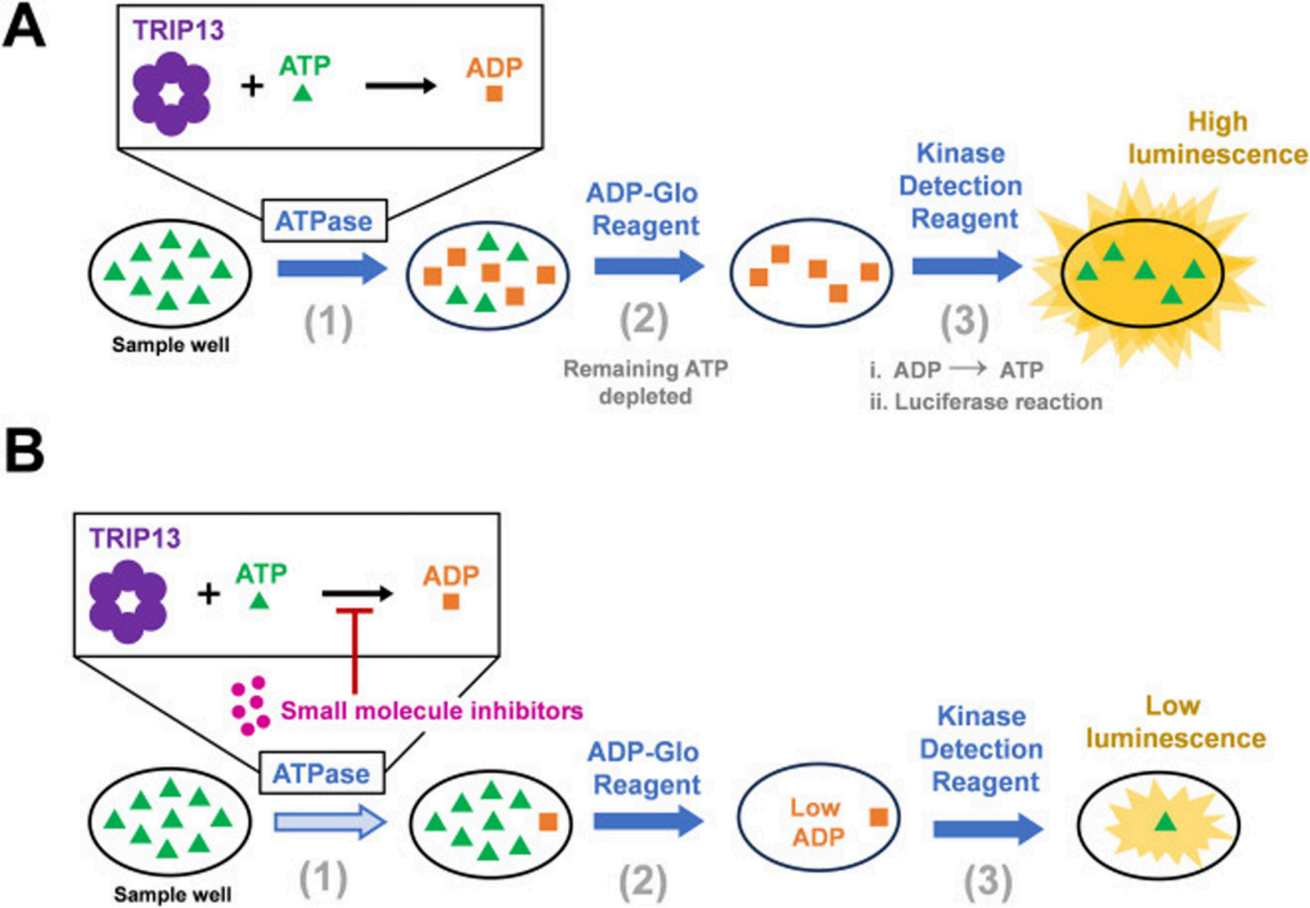
Illustration of Luminescence-based ATPase assay using ADP-Glo Kinase Assay detection system. (A) First, TRIP13 ATPase converts ATP to ADP. The ADP-Glo Reagent (Glo 1) is used to end the ATPase reaction and deplete remaining ATP. Finally, the Kinase Detection Reagent (Glo 2) converts the ADP product from the TRIP13 reaction back into ATP, which produces luminescence signal when processed by luciferase. (B) Small molecule inhibitors reduced ATPase activity, resulting in a decreased luminescence signal.

Successful HTS starts with characterizing optimum assay conditions and developing a cost-effective screening workflow. This study aimed to characterize the activity of TRIP13 using a luminescence-based assay to measure ATPase activity (Fig. 1A) and screen small molecule libraries. The ATPase reaction converts ADP produced by TRIP13 during ATP hydrolysis into a luminescent signal. Therefore, decreased luminescence signifies reduced ATPase activity by the small molecule inhibitors (Fig. 1B). While this method has been used previously to assess the activities of TRIP13 inhibitors [22–24], the assay conditions were diverse, implying a need for optimization and standardization. Specifically, past studies employed high concentrations of TRIP13 (i.e., 1 μM) [24] and ATP (i.e., 100 or 250 μM) [22,23] or were conducted at 37 °C [23], which are not ideal conditions for HTS. The critical considerations for HTS-compatible assay design are robustness, time, and cost-effectiveness.

**Fig. 2.**
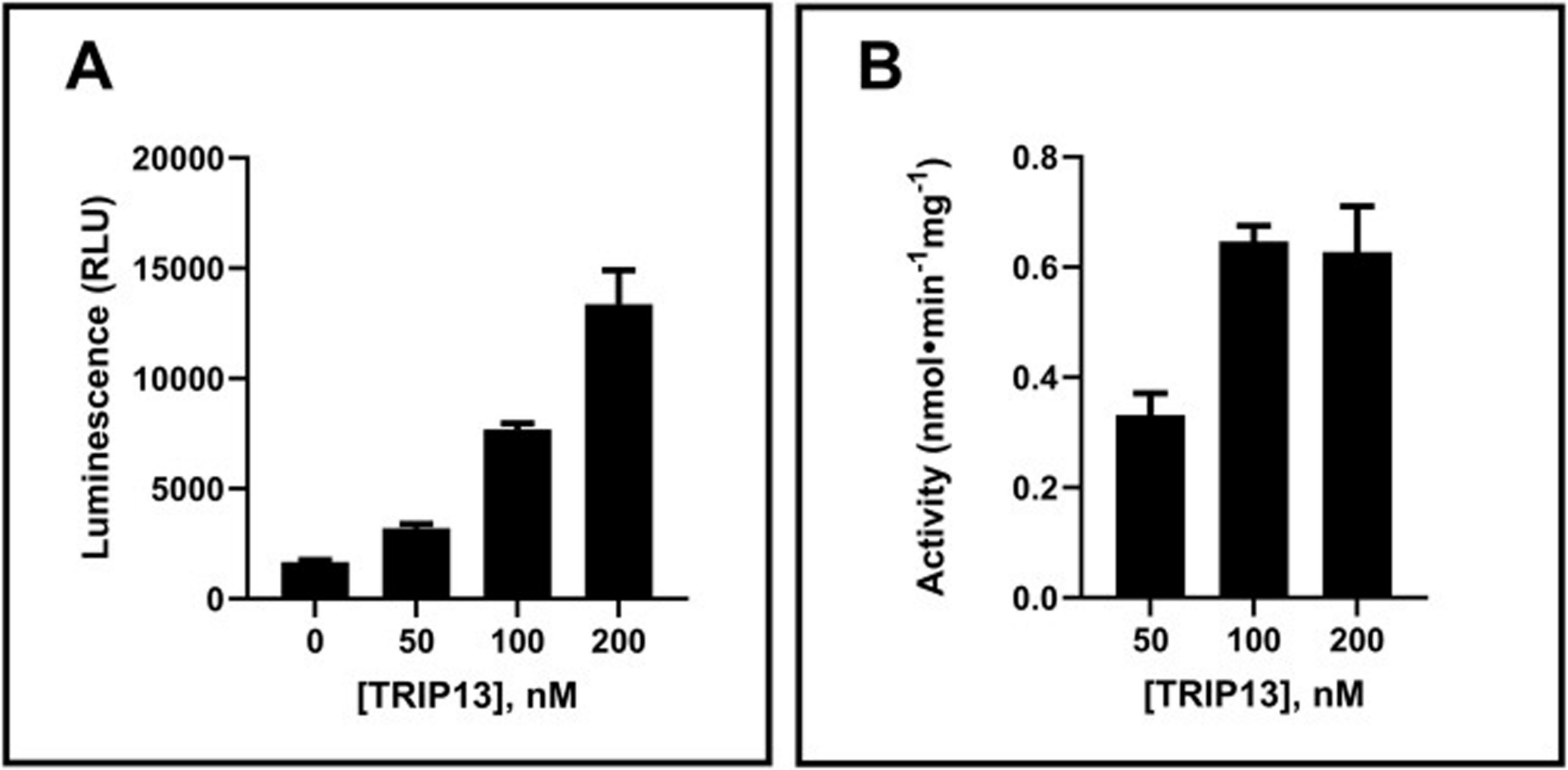
Dose-dependent ATPase activity of TRIP13. TRIP13 (0, 50, 100, and 200 nM) was assayed with 10 μM ATP for 90 min. The resulting relative luminescence signals (A) are also shown in units of enzyme activity (B). Error bars represent the standard deviation of the mean for two replicate samples.

Initially, we characterized the activity of commercially available recombinant full-length TRIP13 (TRIP13) enzyme in the assay at 37 °C for 90 min. TRIP13 exhibited increased luminescence with higher enzyme concentrations (Fig. 2A). To determine the suitability of various TRIP13 constructs, we examined a truncated version (TRIP13-TR) that includes the primary portion of the large AAA domain. This truncated construct is anticipated to preserve ATPase activity. However, TRIP13-TR exhibited two-fold lower activity (data not shown) than full-length TRIP13, confirming that full-length TRIP13 is more suitable for HTS applications. Therefore, we proceeded with full-length TRIP13 for further assay optimization. The dose-dependent luminescence signal was converted to units of enzyme activity to reflect ATP production per min per mg of TRIP13 (Fig. 2B). TRIP13 activity remained consistent over 100 nM, although it displayed lower activity at the lowest concentration assessed (50 nM). As previously reported, this reduced activity at lower concentrations might be attributed to the potential dissociation of the TRIP13 hexamer [22]. Consequently, 100 nM was chosen as the optimal concentration for full-length TRIP13 for further assay optimization.

**Fig. 3.**
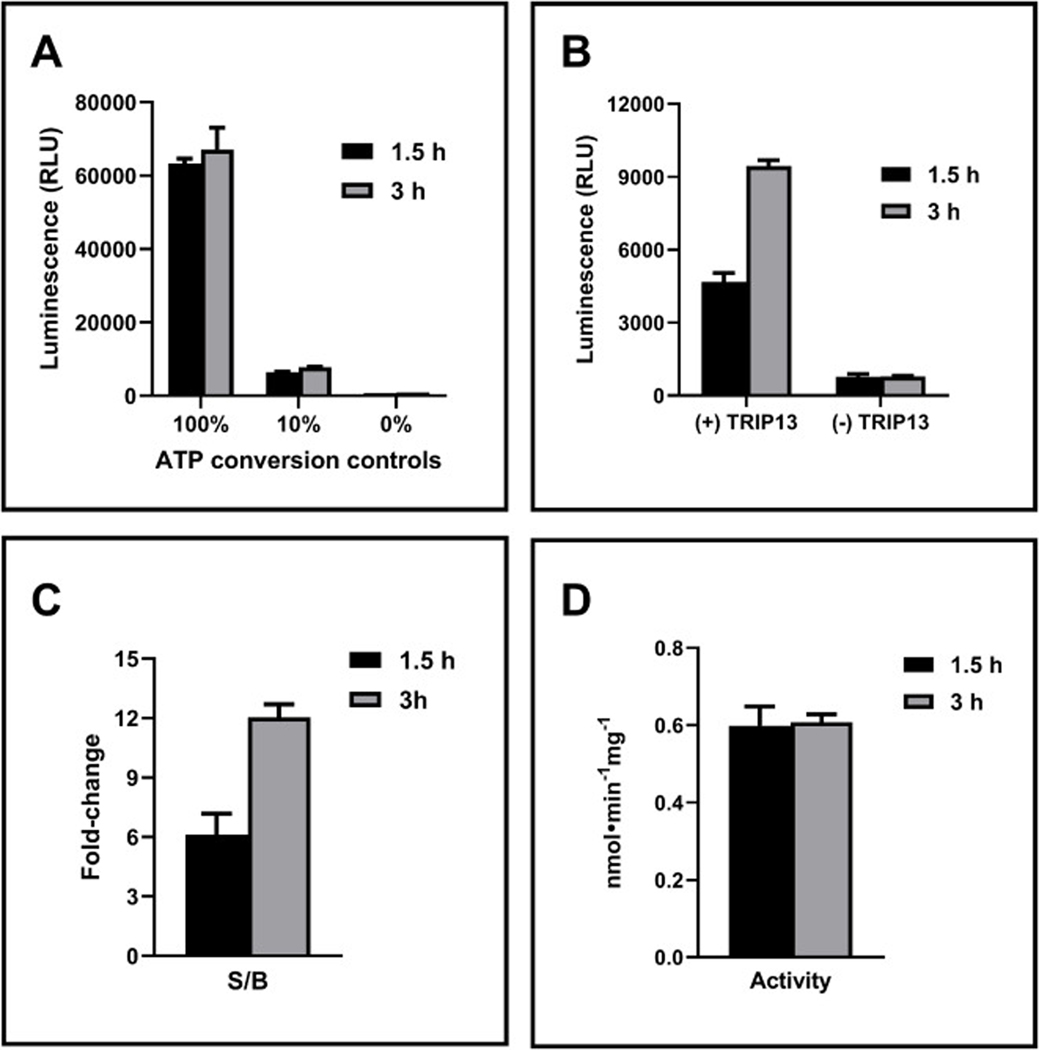
Time-dependent assay performance. (A) Stability of ADP-Glo detection system tested in varying ratio of ATP/ADP representing 0–100 % conversion of 10 μM ATP to ADP. (B) Luminescence signal of TRIP13 at 100 nM (negative control, (+)TRIP13) or buffer only ((−)TRIP13, excluding TRIP13) with 5 μM ATP. (C) Signal-to-background ratio of (+)TRIP13 vs. (−) TRIP13. (D) Activity represents ATP production in nmol per assay time of min per mg of TRIP13. Black and grey filled bars represent 1.5 and 3 h reaction at room temperature, respectively. Error bars represent standard deviation of the mean for two replicate samples.

HTS typically operates at room temperature for streamlining. Therefore, we investigated TRIP13 activity at room temperature for extended durations (up to 3 h). As expected, control experiments (without enzyme) demonstrated a stable signal throughout the 3 h (Fig. 3A), confirming the stability of the ADP-Glo detection system within this timeframe. Encouragingly, the luminescence signal from the TRIP13 reaction increased proportionally with incubation time, as shown by measurements taken at 1.5 h and 3 h, while background luminescence remained constant (Fig. 3B). These conditions resulted in a steadily rising S/B ratio over time (Fig. 3C). Since consistent TRIP13 activity (Fig. 3D) and a high S/B ratio are crucial for HTS applications, a 3 h assay duration at room temperature was chosen as the optimal condition.

**Fig. 4.**
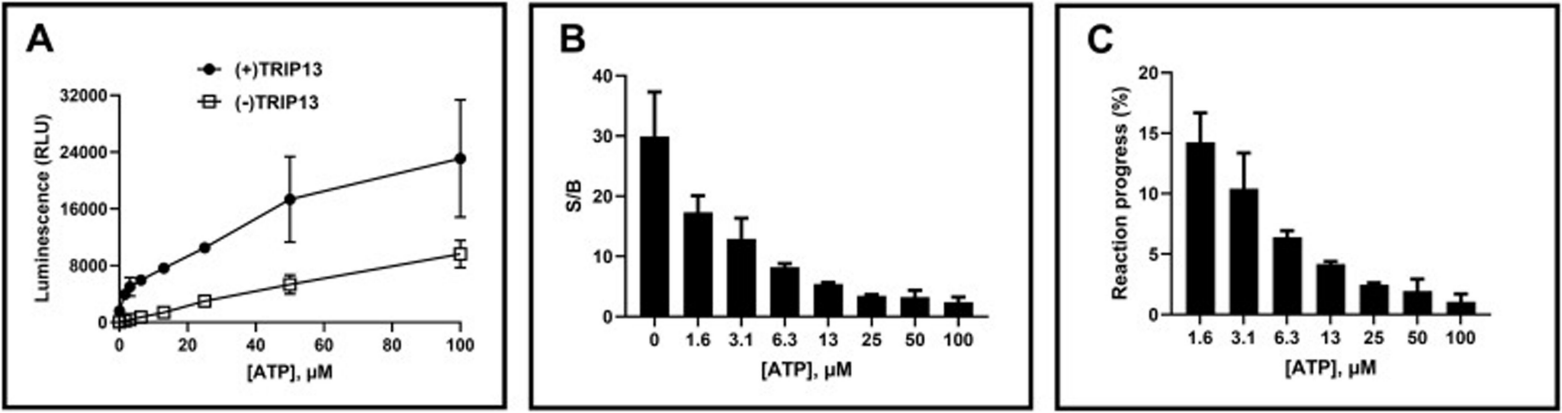
Optimization of ATP substrate. TRIP13 at 100 nM, (+) TRIP13, was assayed with varying concentrations of ATP for 3 h at room temperature. A positive control excluding TRIP13, (−) TRIP13, was assayed in parallel. Luminescence signal (A), signal-to-background ratio (S/B) (B), and reaction progress ( % conversion of ATP to ADP) (C) were monitored. S/B ratio was calculated by dividing negative control luminescence with positive control. Error bars represent the standard deviation of the mean for two replicate samples. Reaction progress was calculated based on controls representing 10 % conversion of ATP to ADP at each ATP concentration.

Next, we optimized the concentration of ATP, the substrate for TRIP13. Various ATP concentrations were evaluated, and the luminescence signal was monitored. A control assay lacking TRIP13 (containing only ATP in assay buffer) was run in parallel to assess background luminescence. Increasing ATP concentration resulted in a stronger luminescence signal (Fig. 4A). However, this was accompanied by a proportional rise in background signal (Fig. 4A). Consequently, the S/B ratio (Fig. 4B) and the percentage of ATP conversion by TRIP13 (Fig. 4C) decreased. Considering the desired parameters for HTS applications are typically a ~10 % conversion rate and an S/B ratio >3, 5 μM was selected as the optimal concentration for ATP. This concentration effectively balanced signal strength with minimal background interference, ensuring reliable detection of TRIP13 activity in subsequent experiments.

**Fig. 5.**
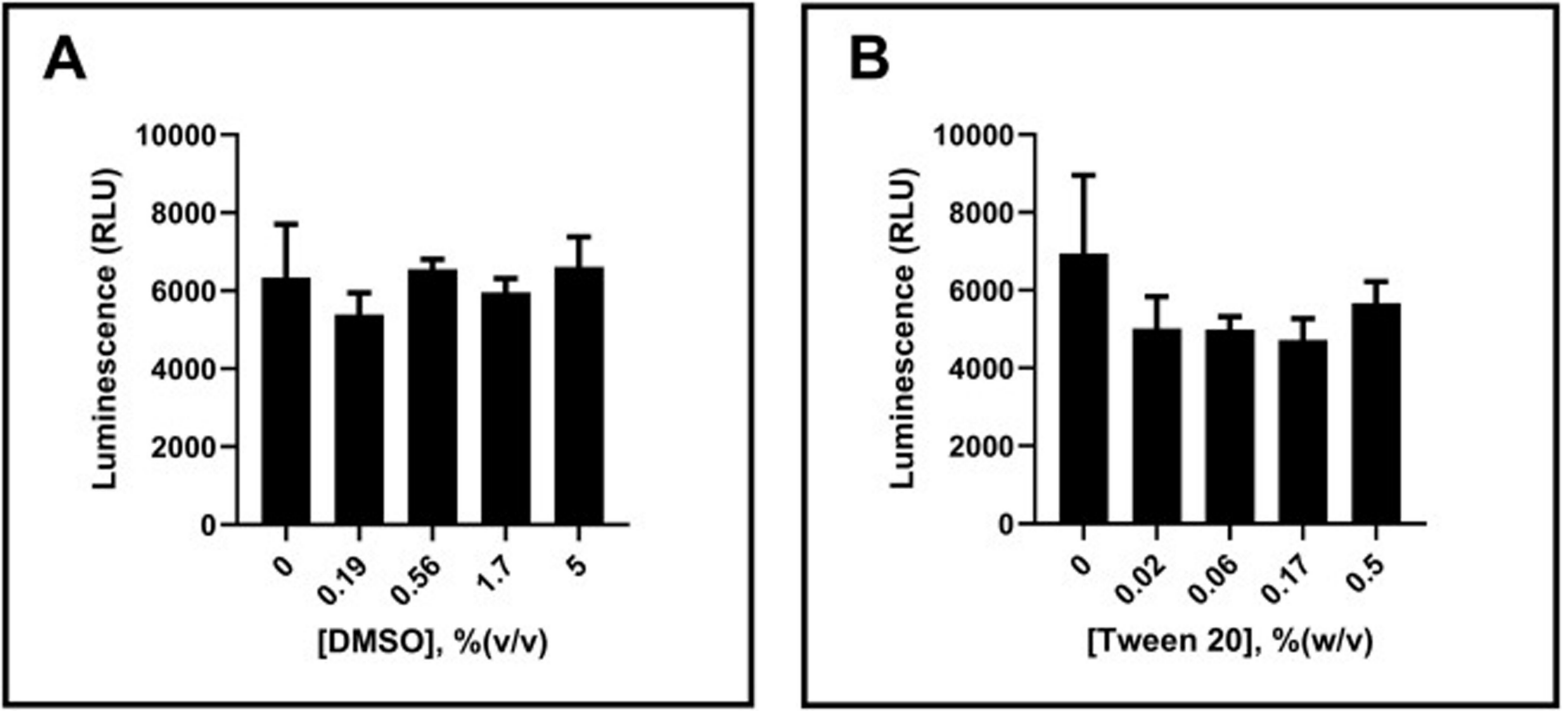
Assay tolerance against DMSO and Tween 20. TRIP13 at 100 nM was assayed with varying concentrations of DMSO (A) and Tween 20 (B) for 3 h at room temperature initiated by 5 μM ATP. Error bars represent the standard deviation of the mean for two replicate samples.

In HTS, small molecule libraries are often dissolved in 100 % DMSO for better solubility and stability. Additionally, HTS assay buffers typically contain detergents to minimize non-specific binding to plastic surfaces, which can lead to inconsistent results. Therefore, assessing the assay’s tolerance to these components is crucial. We evaluated the effects of DMSO and the detergent, Tween 20, on the assay performance (Figs. 5A and 5B). The results indicate that the assay tolerates up to 5 % (v/v) DMSO and 0.5 % (w/v) Tween 20 without compromising enzyme activity. This result ensures that the DMSO amount at the chosen testing concentration of 10 μM for compounds will not interfere with the enzyme activity. Additionally, the typical concentration of 0.02 % (w/v) Tween 20, commonly used in HTS campaigns, was confirmed to be compatible with the assay’s tolerance range. This Tween 20 concentration was pre-selected based on its established use in previous HTS studies [30].

To minimize reagent costs, we investigated the possibility of reducing the volume of ADP-Glo Kinase Assay kit used in the assay. The original protocol from Promega calls for a volumetric composition of one part sample mixture, one part Glo 1, and two parts Glo 2. We examined the assay performance by reducing the Glo 1 and Glo 2 vol in various increments. We found that a 50 % reduction in both the Glo 1 and Glo 2 vol did not compromise assay quality or sensitivity (data not shown). This optimization effectively reduces the cost of ADP-Glo Kinase Assay kit twofold.

### 3.2. Assay validation and compound screening

To assess the assay’s performance before moving on to small molecule libraries, we tested a validation plate using DMSO at a concentration of 0.1 % (v/v), a solvent concentration mimicking the presence of a 10 μM test compound. We processed the validation plate as illustrated in Figure S1A. This validation run yielded a z’-factor of 0.85 and a S/B of 5.75, indicating excellent assay robustness for HTS. A further evaluation of the assay’s ability to detect TRIP13 inhibitors was conducted by testing DCZ0415 [21], the first reported inhibitor of TRIP13. A previous study reported a weak activity for DCZ0415, with the IC_50_ of 57 μM determined by SPR, and minimal activity observed in an ATPase activity using 1 μM of His-tagged TRIP13 [24]. Notably, the inhibitor concentration used in the previous study was not disclosed. While DCZ0415 did not exhibit inhibitory activity up to 500 μM in our assay (data not shown), the assay’s robust performance demonstrated by the validation run provides confidence in its suitability for compound screening.

**Fig. 6.**
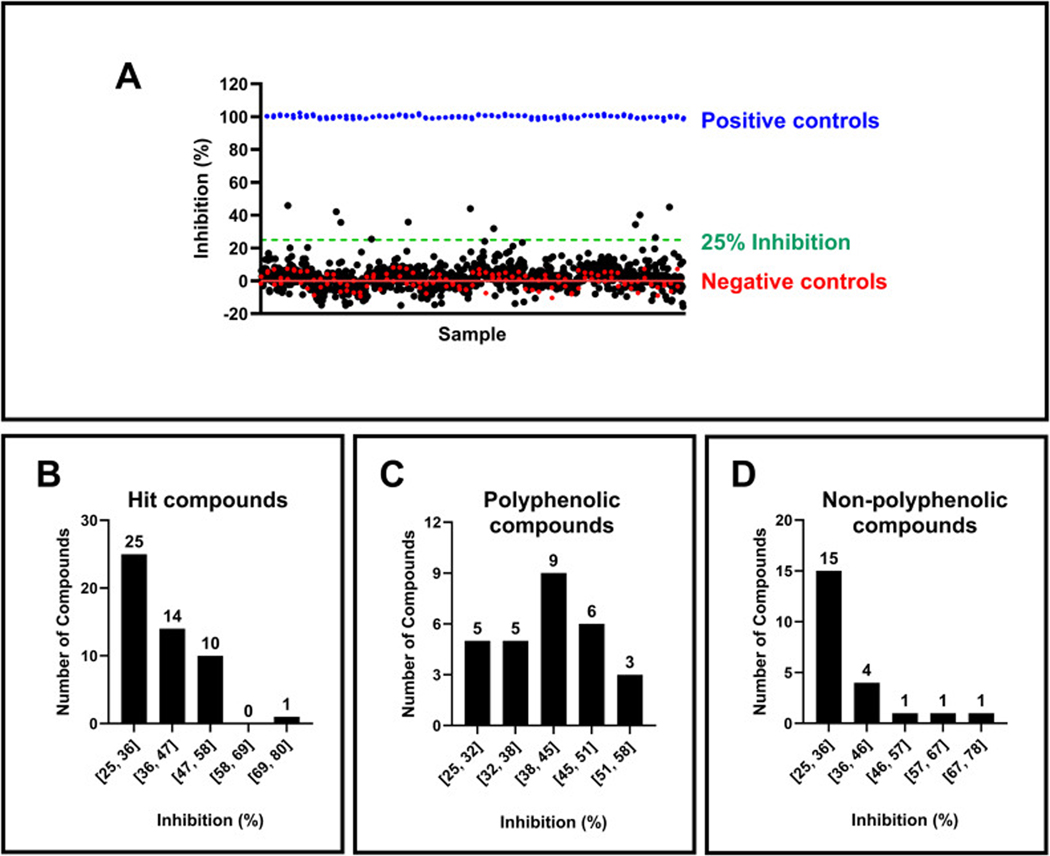
Scatter plot of pilot screening and histograms of hit compounds activity. (A) Representative scatter plot of pilot screening. The blue data points represent positive (100 % inhibition) controls. Red data points indicate negative (0 % inhibition) controls, with a red line at inhibition = 0 %. Black data points indicate samples with library compounds. In comparison, the green dashed line represents the hit threshold of 25 % inhibition. (B) The distribution of hit compounds from both pilot and primary screenings. (C) The distribution of hit compounds with polyphenolic structures. (D) The distribution of hit compounds with non-polyphenolic structures. X-axis and Y-axis represent the range of % inhibition and number of compounds, respectively, in (B), (C), and (D).

Before proceeding with a large-scale screen, a pilot screen on a smaller scale was conducted to further confirm the assay’s suitability for the HTS procedure and data analysis. This pilot employed a set of approximately 1000 compounds custom-curated with known activity against various kinases. These compounds were assayed in single replicates at 10 μM concentration across four assay plates. An example library plate layout is shown in Figure S1B and the assay plate layout is shown in Figure S1C. The pilot screen demonstrated a clear separation between positive and negative controls (Fig. 6A) with an average z’-factor of 0.84 ± 0.01 and an S/B ratio of 5.54 ± 0.17. The excellent assay statistics indicated high reproducibility and confirmed the assay’s suitability for large-scale screening.

Encouraged by the pilot’s success, a large-scale screen (i.e., primary screen) was conducted using a custom-curated bioactive screening library containing approximately 4000 small molecules with confirmed biological activities and clear targets. These compounds are structurally diverse and have been tested in functional, binding, and other biological assays. They include natural products, FDA-approved compounds, and active pharmaceutical and chemotherapeutic agents. These compound sets provide useful tools for signal pathway research, drug discovery, and drug repurposing. These compounds were assayed in single replicates at 10 μM concentration across sixteen assay plates. The assay performed excellently, with an average z’-factor of 0.85 ± 0.01 and an S/B ratio of 5.83 ± 0.25. The pilot and primary screens identified 50 initial hits exhibiting over 25 % inhibition (Fig. 6B). Interestingly, most hits displayed weak to moderate inhibitory activity (25 – 50 %), and over half (28 compounds) of the hits are polyphenolic acids (Fig. 6C). These findings suggest the potential for a high rate of non-specific hits or therapeutic impact of polyphenols on TRIP13 stability and function. Several studies have reported on the protein-binding properties of polyphenols and their ability to elicit enzyme inhibition [31–33]. Notably, hits with polyphenolic structures exhibited a broader range of inhibition activities than non-polyphenolic compounds (Figs. 6C and 6D), which emphasized further study to elucidate the mechanism of action.

### 3.3. Hit validation via a series of biochemical and cellular assays

The initial hit validation employed two key strategies: rescreening and counter-screening. All identified hits were retested at 10 μM to assess the reproducibility of their inhibitory activity. The counter-screening step aimed to identify compounds that might interfere with the assay rather than truly inhibiting TRIP13 activity. To achieve this, the counter-screening assay used 0.5 μM ADP in place of the TRIP13/ATP mixture. This concentration of ADP was chosen because it generates a signal equivalent to the negative control, corresponding to approximately 10 % conversion of ATP to ADP by TRIP13 in the primary assay. Compounds exhibiting similar activity in the primary and counter screens are more likely to interfere with the detection reagents than to inhibit the enzyme.

**Fig. 7.**
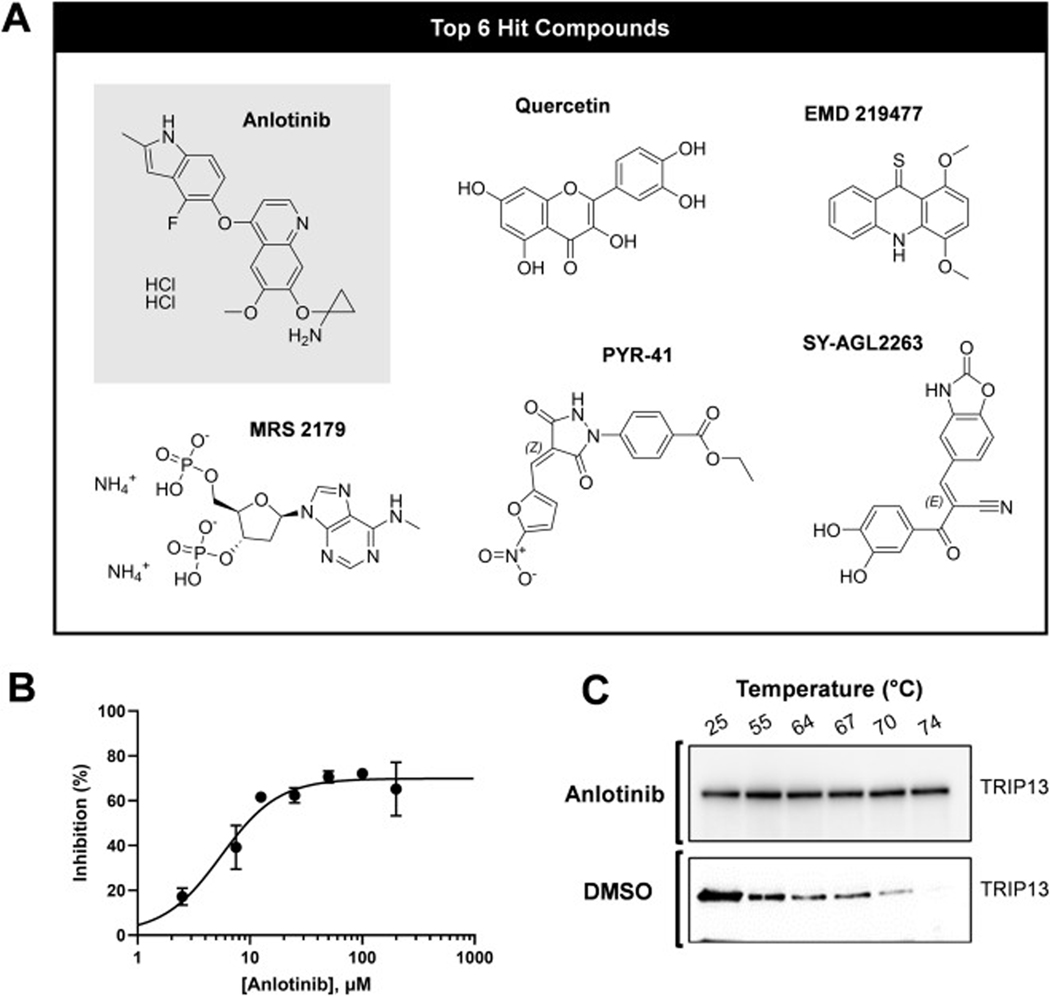
Hit molecule characterization. (A) Chemical structures of the 6 hit compounds. Anlotinib is highlighted in gray. (B) Dose responsive activity profile of anlotinib performed with 150 nM TRIP13. Data points were fit to the four-parameter logistic model from SigmaPlot, resulting in IC_50_ and Hill parameter of 5.5 ± 1.03 μM and 1.69 ± 0.43, respectively. Error bars represent the standard deviation of the mean for two replicate samples. (C) Western blot of each analyzed TRIP13 in the soluble fraction from HeLa cell lysate after temperature exposure in the presence of anlotinib (20 μM) and DMSO.

Following rescreening and counter-screening at 10 μM, six compounds shown in Fig. 7A (anlotinib, quercetin, PYR-41, MRS 2179, EMD 219,477, SY-AGL2263) displayed reproducible inhibitory activity in the rescreen and exhibited minimal activity (<10 %) in the counter-screening assay, suggesting they may be selectively targeting TRIP13 rather than interfering with the assay itself. These six compounds were, therefore, selected for potency evaluation to determine their concentration-dependent inhibitory effect on TRIP13 activity (Figure S2), resulting in anlotinib as the most potent inhibitor with IC_50_ of 5.5 ± 1.0 μM (Fig. 7B).

To further validate whether anlotinib targets TRIP13, we investigated the binding activity of anlotinib to TRIP13 at the cellular level using the Cellular Thermal Shift Assay (CETSA). As shown in Fig. 7C, anlotinib increased the thermal stability of TRIP13 compared to DMSO in HeLa cells, confirming a specific physical interaction between anlotinib and TRIP13 within the cellular environment. These results provide compelling support for the inhibition of anlotinib via direct interaction with TRIP13.

## 4. Conclusion

This study successfully developed and optimized a cost-effective HTS-compatible assay for TRIP13 activity using the ADP-Glo detection system. Following optimization, the luminescence-based assay demonstrated excellent performance with an S/B ratio greater than 5.5 and a robust z’-factor exceeding 0.8, qualifying its capability for HTS applications. Through a pilot screen and a subsequent large-scale screen of 4000 compounds, 50 initial hits were selected for further investigation. Rigorous validation, including rescreening, counter-screening, and potency evaluation, narrowed the hits to six promising candidates. Anlotinib was identified as the most potent inhibitor of TRIP13 from the screen, and its direct binding to TRIP13 was further confirmed by CETSA.

One limitation of our study is that we used a kinase inhibitor library for our initial drug screen to identify inhibitors of an AAA+ ATPase. Anlotinib inhibits c-kit, platelet-derived growth factor receptors, fibroblast growth factor receptors, and vascular endothelial growth factor receptors [34]. While anlotinib is not a specific TRIP13 inhibitor, this finding highlights the potential for repurposing existing drugs or identifying novel drug scaffolds with dual or multi-target activity against TRIP13 and other cancer-relevant targets. Future drug screens using our HTS strategy will include a broader range of drugs to identify more specific inhibitors.

We will also test future drug candidates for specificity by evaluating their effects on other AAA+ ATPases. The AAA+ ATPase (AAA+) superfamily members are categorized into 34 protein families based on the presence of unique structural elements within and around the core AAA+ fold [35,36] and fall into eight major categories depending on their cellular activities, which consist of ATP-dependent proteases, membrane fusion proteins, peroxins (Pex1p and Pex6p), katanin and SKD1/Vps4p, dynein, DNA replication proteins, RuvB, and Eukaryotic RuvB-like proteins [37]. There are 149 identified human AAA+ ATPases, including TRIP13 [38]. Phylogenetic analysis of TRIP13 and its ortholog Pch2 based on the structure and sequence comparison of the conserved AAA+ ATPase module and substrate recognition domain, revealed a significant divergence between Pch2/TRIP13 and other members of the AAA+ superfamily, indicating that Pch2/TRIP13 is unique [39]. The distinct structure of TRIP13 may lead to identifying specific inhibitors.

This optimized HTS assay paves the way for discovering novel, potent, and selective TRIP13 inhibitors, ultimately contributing to developing affordable new therapeutic strategies for Rb-deficient and other cancers.

## Supplementary Material

Supporting Information

Supplementary material associated with this article can be found, in the online version, at doi:10.1016/j.slasd.2025.100233.
