# Supplementary material for: Advancing the development of TRIP13 inhibitors: A high-throughput screening approach
Source: SLAS Discov. Author manuscript; Available in PMC 2025 Jul 3. (PMC12224672; doi:10.1016/j.slasd.2025.100233)
Supplement: Supporting Information [file NIHMS2082824-supplement-Supporting_Information.docx]

**Supporting Information**

**Advancing the Development of TRIP13 Inhibitors: A High-Throughput Screening Approach**

Rae M. Sammons,^1†^ Soma Ghosh,^2^ Lacin Yapindi,^2^ Eun Jeong Cho,^1†*^ Faye M. Johnson,^2^ and Kevin N. Dalby^1,3*^

^1^Targeted Therapeutic Drug Discovery & Development Program, The University of Texas at Austin, Austin, TX

^2^Thoracic, Head and Neck Medical Oncology, The University of Texas, MD Anderson Cancer Center, Houston, TX

^3^Division of Chemical Biology & Medicinal Chemistry, College of Pharmacy, The University of Texas at Austin, Austin, TX

*Corresponding authors (These authors contributed equally to this work): Kevin N. Dalby (dalby@austin.utexas.edu) and Eun Jeong Cho (euncho@austin.uteas.edu)

^†^ These authors are co-first authors: Rae M. Sammons and Eun Jeong Cho

**Contents**:

**Figure S1**

Illustration of HTS workflow, library plate layout, and assay plate map S-2

**Figure S2**

Dose-dependent inhibition of TRIP13 activity by the top 6 hit compounds S-3

**
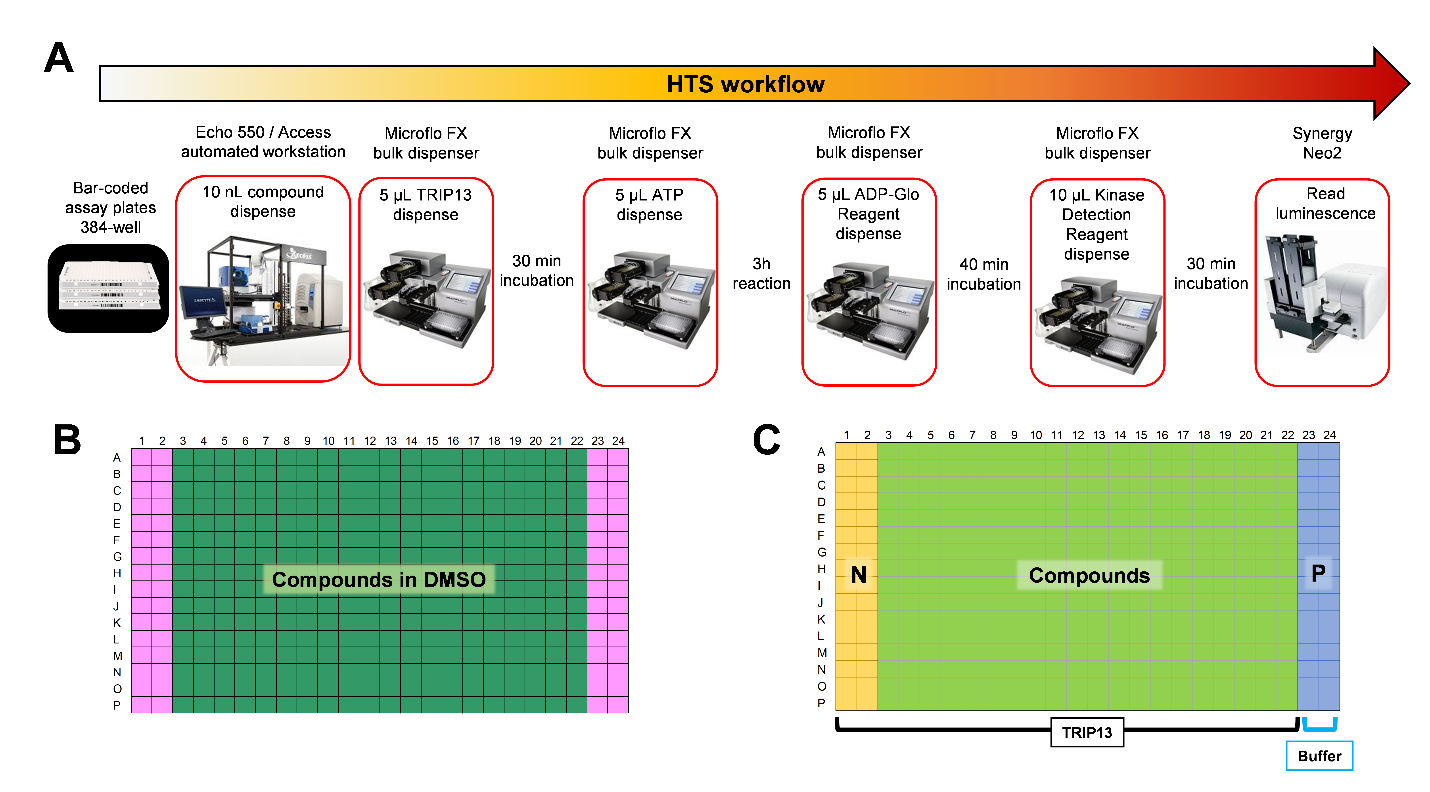
Figure S1. Illustration of HTS workflow, library plate layout, and assay plate map.**

(A) Automated HTS starts with compound dispensing to a dry assay plate using Echo 550, then dispensing TRIP13 or buffer using a Microflo FX. After 30 min incubation, substrate and detection reagents are dispensed serially after designated incubation times before reading on a plate reader. (B) Compounds are dispensed on assay plates between columns 3-22 (green), while DMSO is dispensed on columns 1, 2, 23, and 24 (pink). (C) TRIP13 or buffer is dispensed to columns 1-22 or 23-24, respectively, followed by ATP dispensing to the entire plate. Consequently, columns 1-2 are negative control (N, yellow), and columns 23-24 are positive control (P, blue) for data analysis.


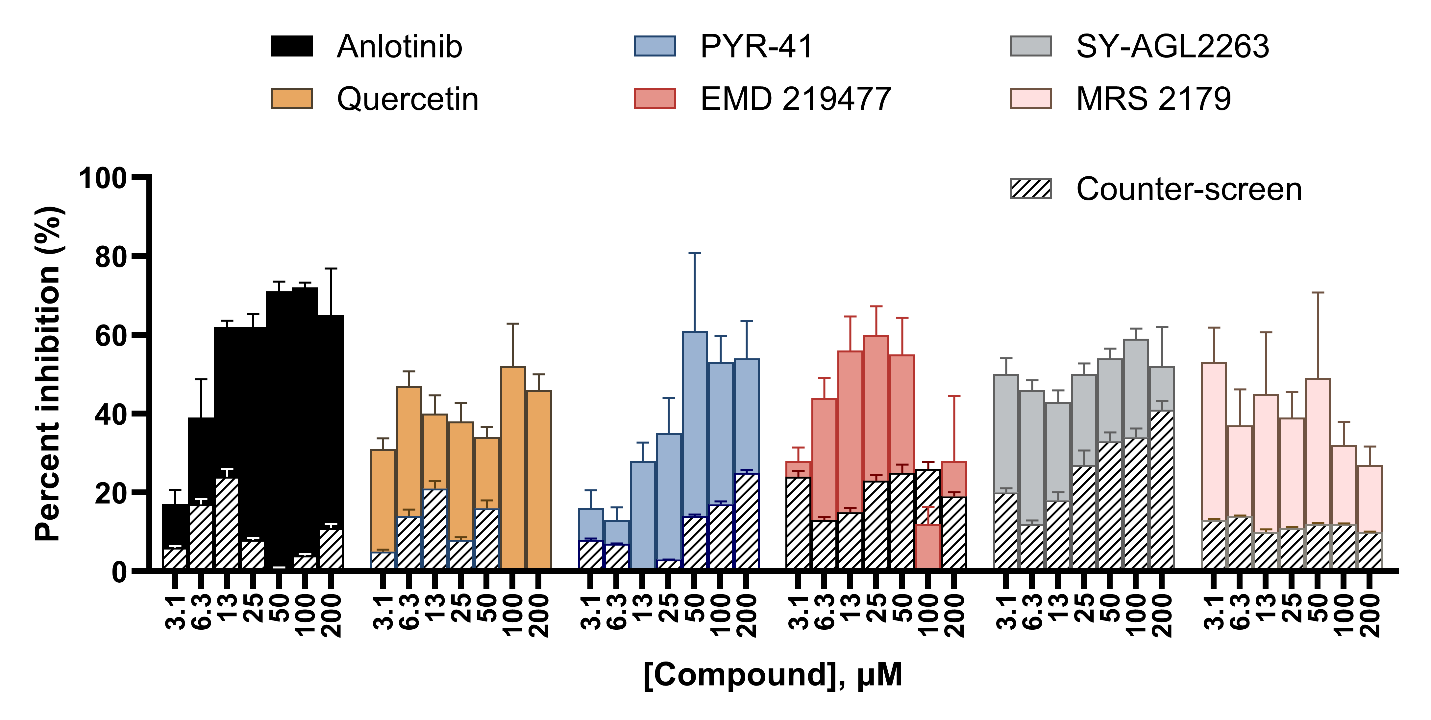


**Figure S2. Dose-dependent inhibition of TRIP13 activity by the top 6 hit compounds.**

The top 6 compounds (anlotinib, quercetin, PYR-41, EMD 219477, MRS 2179, and SY-AGL2263) were tested at concentrations of 0-200 µM for their ability to inhibit TRIP13 activity in the presence of 5 µM ATP. For counter-screen controls, the same concentrations of each inhibitor were tested in the presence of 0.5 µM ADP to identify any dose-dependent interference in the ADP-Glo reagents. Percent inhibition indicates inhibition (%) of signal in the activity assay, normalized to DMSO controls.
